# Association of Prognostic Nutritional Index and Mortality in Older Adults Undergoing Hip Fracture Surgery: A Retrospective Observational Study at a Single Large Center

**DOI:** 10.3390/medicina61081376

**Published:** 2025-07-30

**Authors:** Yeon Ju Kim, Ji-In Park, Hyungtae Kim, Won Uk Koh, Young-Jin Ro, Ha-Jung Kim

**Affiliations:** 1 Department of Anesthesiology and Pain Medicine, Asan Medical Center, University of Ulsan College of Medicine, 88 Olympic-ro 43-gil, Songpa-gu, Seoul 05505, Republic of Korea; yjans@amc.seoul.kr (Y.J.K.);; 2 Department of Anesthesiology and Pain Medicine, Seoul National University Bundang Hospital, Seongnam 13620, Republic of Korea

**Keywords:** prognostic nutritional index, mortality, hip fractures

## Abstract

*Background and Objectives*: Patients with hip fractures have a high mortality rate, highlighting the need for a reliable prognostic tool. Although the prognostic nutritional index (PNI) is a well-established predictor in patients with cancer, its utility has not been thoroughly investigated in patients with hip fractures. Therefore, this study aims to evaluate the association between PNI and mortality in patients undergoing hip fracture surgery. *Materials and Methods*: A retrospective review was conducted on all patients aged ≥65 years who underwent surgery for hip fracture between January 2014 and February 2018. Quartile stratification was chosen because no universally accepted clinical cut-off exists for PNI; this approach enables comparison of equally sized groups and exploration of potential non-linear risk patterns. The primary endpoints were 1-year and overall mortality in older adults undergoing hip fracture surgery. Multivariable Cox proportional-hazards models adjusted for age, sex, ASA class and comorbidities. *Results*: A total of 815 patients were analyzed. One-year and overall mortality rates were highest in the Q1 group (26.6%, 14.2%, 6.9%, 6.4% [*p* < 0.001] and 56.7%, 36.3%, 27.0%, 15.2% [*p* < 0.001], respectively). In Cox regression analysis, a lower preoperative PNI was significantly associated with an increased risk of overall mortality (Q1: HR 3.25, 95% confidence interval [CI] 2.11–5.01, *p* < 0.001; Q2: HR 1.85, 95% CI 1.19–2.86, *p* = 0.006; Q3: HR 1.52, 95% CI 0.97–2.38, *p* = 0.065; Q4 as reference), indicating a stepwise, dose–response increase in mortality risk as PNI decreases. *Conclusions*: The findings demonstrate that a lower preoperative PNI is significantly associated with higher 1-year and overall mortality in older adults undergoing hip fracture surgery. Although further prospective validation is needed, preoperative PNI may help predict mortality in frail patients undergoing hip fracture surgery and identify those who could benefit from nutritional assessment and optimization before surgery.

## 1. Introduction

Older adults with hip fractures experience a 30% mortality rate within the following year, and many sustain long-term functional decline [1,2]. This high risk is largely attributed to the demographic characteristics of this population, which primarily consists of frail individuals with multiple comorbidities and compromised nutritional status [3]. In addition, age-related physiological changes introduce unique postoperative challenges that further impact recovery. Accordingly, comprehensive assessment of morbidity and mortality risk, along with careful perioperative management, is essential to improve clinical outcomes following surgery.

Malnutrition contributes to approximately 50% of admissions in patients with hip fractures and is widely recognized as a major factor associated with poor postoperative outcomes [4,5] due to its detrimental effects on immune function and increased susceptibility to infection [6]. Various tools have been employed to evaluate nutritional status and predict mortality, including the Controlling Nutritional Status, Geriatric Nutritional Risk Index, and Mini Nutritional Assessment-Short Form [7]. However, these tools require direct responses from patients, which may be impractical for older adults with cognitive impairment.

The prognostic nutritional index (PNI), calculated from serum albumin and lymphocyte count, offers a practical alternative. It correlates with postoperative morbidity and mortality in patients with cancer [8,9,10]. Serum albumin reflects both nutritional status and systemic inflammation, while lymphocyte count serves as an indicator of immune competence. Together, these markers provide an objective measure of physiological reserve, which may be particularly relevant in older adults undergoing surgery. Unlike other assessment tools, PNI relies solely on routine laboratory data and can be readily applied in urgent clinical settings such as hip fracture surgery. However, limited studies have assessed the role of PNI in predicting outcomes in patients with hip fractures, and existing data are insufficient to establish its prognostic value in this population [7].

Therefore, this study aims to investigate the association between preoperative PNI and both 1-year and overall mortality in older adults undergoing hip fracture surgery. Additionally, we examined associations between preoperative PNI, length of hospital stay, and rehospitalization within 1 year following discharge. We hypothesized that lower PNI would be independently associated with worse outcomes.

## 2. Materials and Methods

### 2.1. Study Design and Participation

This retrospective study was approved by the Institutional Review Board of Asan Medical Center (IRB No. 2021-0924) on 19 June 2021, and informed consent was waived due to the study’s retrospective nature. We reviewed medical records of all patients aged ≥65 years who underwent surgery for hip fracture between January 2014 and February 2018 to investigate the impact of PNI on postoperative mortality rates. Patients were excluded if preoperative PNI data were missing or if they had undergone surgery for the contralateral side hip.

To evaluate the association between PNI and postoperative outcomes, patients were categorized into quartiles based on preoperative PNI in the final analysis.

### 2.2. Clinical Data Collection and Definitions

Baseline demographic data, laboratory results, and postoperative outcomes—including mortality, length of hospital stay, and readmission within 1 year—were extracted from the electronic medical record system of Asan Medical Center. Demographic variables included age, sex, weight, height, body mass index (BMI), and the American Society of Anesthesiologists (ASA) physical status classification.

Preoperative laboratory data included serum albumin, hemoglobin (Hb), creatinine, and C-reactive protein (CRP). Hypoalbuminemia was defined as serum albumin <3.5 g/dL. Anemia was defined as Hb < 12 g/dL in females and Hb < 13 g/dL in males. The PNI was calculated using the following formula: [10 × serum albumin (g/dL)] + [0.005 × total lymphocyte count (/mm^3^)] [11].

Additional clinical information included the type of surgery, anesthesia method, and operation time. Surgeries performed within 48 h of hospital admission were classified as early surgery; those performed after 48 h were categorized as late surgery. Readmission was defined as any unplanned hospitalization to our institution within 365 days following the index discharge. Postoperative complications included cardiovascular, pulmonary, neurological, and renal complications. Delirium was evaluated separately and diagnosed by a psychiatrist according to DSM-5 criteria. Postoperative outcomes included 1-year mortality (from the date of surgery to the 1-year follow-up), overall mortality (from the date of surgery to the most recent follow-up), length of hospital stay, and readmission for any cause within 1 year.

### 2.3. Primary and Secondary Outcomes

The primary outcomes were 1-year and overall mortality in older adults undergoing hip fracture surgery. Multivariable analysis was conducted to identify factors associated with mortality. Secondary outcomes included length of hospital stay, readmission within 1 year, and postoperative complications (including cardiovascular, pulmonary, and neurologic events; acute kidney injury; and delirium).

### 2.4. Statistical Analysis

Continuous variables were expressed as means with standard deviations or as medians with interquartile ranges, while categorical variables were reported as frequencies (%). Continuous variables were compared using analysis of variance, and categorical variables were compared using the chi-square test.

To guide PNI-based stratification, receiver-operating characteristic (ROC) analysis was conducted to predict overall mortality. The area under the curve was 0.70 (95% CI 0.66–0.74), and the optimal threshold derived using Youden’s index was 39.2. However, to retain granularity and allow exploration of non-linear risk patterns, PNI was ultimately categorized into quartiles.

Kaplan–Meier survival curves were constructed for each PNI quartile group and compared using the log-rank test. Univariable associations with mortality were screened, and variables with a *p*-value < 0.10 or considered clinically relevant a priori (age, sex, BMI, ASA class, CRP, creatinine) were entered into multivariable Cox proportional-hazards models.

The proportional hazards assumption was verified using Schoenfeld residuals, with the global test yielding *p* = 0.42, indicating no significant violation. Missing data accounted for less than 5% of all observations and were presumed to be randomly distributed; therefore, complete-case analysis was performed without imputation.

To ensure adequate statistical power, a post hoc power calculation was conducted. With 815 patients and an observed overall mortality rate of 34%, the study had 80% power (α = 0.05) to detect a hazard ratio ≥1.35 between the lowest and highest PNI quartiles.

All statistical analyses were performed using IBM SPSS Statistics for Windows, version 22.0 (IBM Corp., Armonk, NY, USA), and a *p*-value < 0.05 was considered statistically significant.

## 3. Results

**Figure 1:**
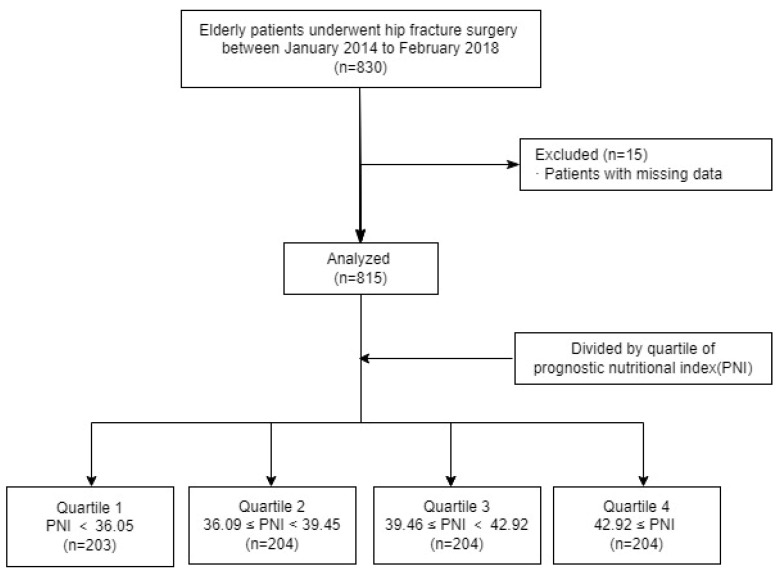
Study flow chart.

Of the 830 enrolled patients, 15 patients with incomplete data were excluded from the analysis. A total of 815 patients were included in the final analysis. Patients were stratified into four groups based on the PNI quartiles: Quartile 1 (PNI < 36.09, n = 203), Quartile 2 (36.09 ≤ PNI < 39.45, n = 204), Quartile 3 (39.46 ≤ PNI < 42.92, n = 204), and Quartile 4 (42.92 ≤ PNI, n = 204) (Figure 1).

Demographic characteristics, preoperative status, postoperative adverse outcomes, postoperative hospital stay duration, readmission within 1 year, and 1-year overall mortality are summarized in Table 1. Patients in the lower preoperative PNI quartiles were more likely to be male, have lower BMI, and present with poorer ASA classification (Table 1). These groups had lower levels of hemoglobin and albumin and higher levels of CRP and creatinine (Table 1).

### Outcomes

Table 2 shows 1-year and overall mortality by PNI quartile. One-year mortality was 26.6% (54/203, 95% CI 21.0–33.1) in the Q1 group, 14.2% (29/204, 95% CI 10.1–19.7) in the Q2 group, 6.9% (14/204, 95% CI 4.1–11.2) in the Q3 group, and 6.4% (13/204, 95% CI 3.8–10.6) in the Q4 group. The overall mortality was 56.7% (115/203, 95% CI 49.8–63.3) in the Q1 group, 36.3% (74/204, 95% CI 30.0–43.1) in the Q2 group, 27.0% (55/204, 95% CI 21.3–33.4) in the Q3 group, and 15.2% (31/204, 95% CI 10.9–20.8) in the Q4 group. In addition, the 1-year standardized mortality rates were 26.0% for Q1, 11.0% for Q2, and 6.4% for Q3, and the overall standardized mortality rates were 53.6% for Q1, 30.0% for Q2, and 23.1% for Q3, respectively.

**Figure 2:**
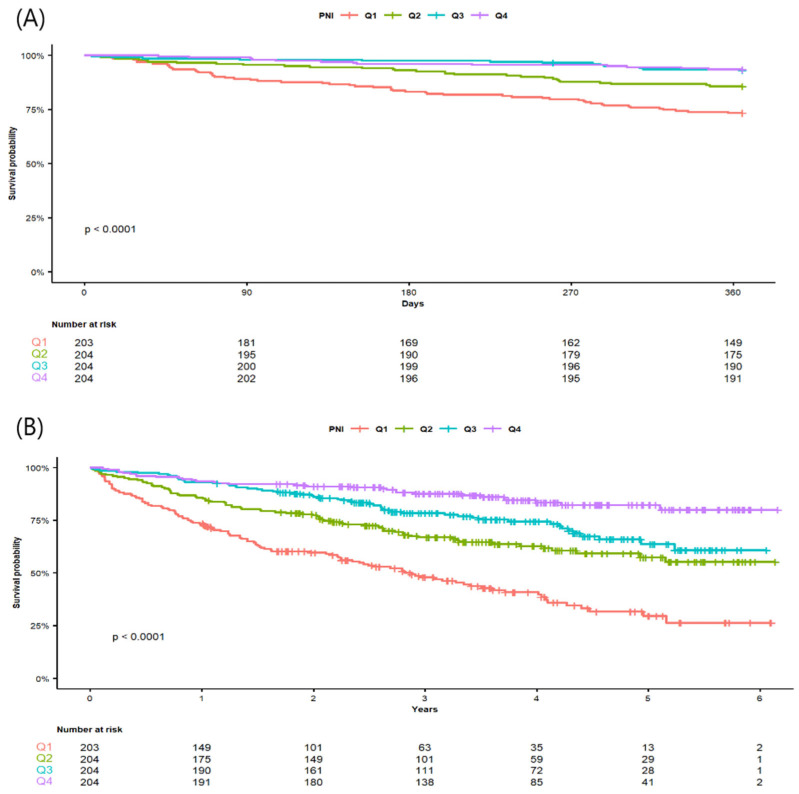
Kaplan–Meier survival curve for (**A**) 1-year and (**B**) overall survival (log-rank test; *p* < 0.001).

Kaplan–Meier estimated survival probability at the end of follow-up was 26.2% (95% CI 16.9–36.5) in Q1, 55.2% (45.6–63.7) in Q2, 60.7% (49.5–70.2) in Q3, and 80.0% (71.4–86.3) in Q4, respectively. Kaplan–Meier survival curves for 1-year and overall survival by PNI quartile are shown in Figure 2. Both 1-year (Figure 2A) and overall survival (Figure 2B) differed significantly among quartiles (log-rank *p* < 0.001).

In the Cox regression analysis, lower preoperative PNI was significantly associated with increased overall mortality risk (Q1: HR 3.25, 95% CI 2.11–5.01, *p* < 0.001; Q2: HR 1.85, 95% CI 1.19–2.86, *p* = 0.006; Q3: HR 1.52, 95% CI 0.97–2.38, *p* = 0.065, Q4 as reference). Additional variables significantly associated with overall mortality included older age (HR 1.04, 95% CI 1.02–1.06, *p* < 0.001), male sex (HR 1.60, 95% CI 1.24–2.06, *p* < 0.001), BMI (HR 0.94, 95% CI 0.91–0.97, *p* < 0.001), diabetes mellitus (DM) (HR 1.54, 95% CI 1.19–2.00, *p* < 0.001), ASA class 3 (HR 1.66, 95% CI 1.29–2.14, *p* < 0.001), ASA class 4 (HR 2.82, 95% CI 1.12–7.06, *p* = 0.027), CRP (HR 1.03, 95% CI 1.00–1.06, *p* = 0.035), and creatinine (HR 1.15, 95% CI 1.05–1.25, *p* = 0.001) (Table 3). Variables such as hypertension, cognitive impairment, and operation type were not independently associated with mortality after multivariate adjustment, suggesting their effects were confounded or explained by stronger predictors in the model. Model fit was adequate, with no evidence of lack of fit. The proportional hazards assumption was confirmed by Schoenfeld residuals (global test *p* = 0.42), and no significant collinearity was detected among covariates (all VIFs < 2.0).

**Figure 3:**
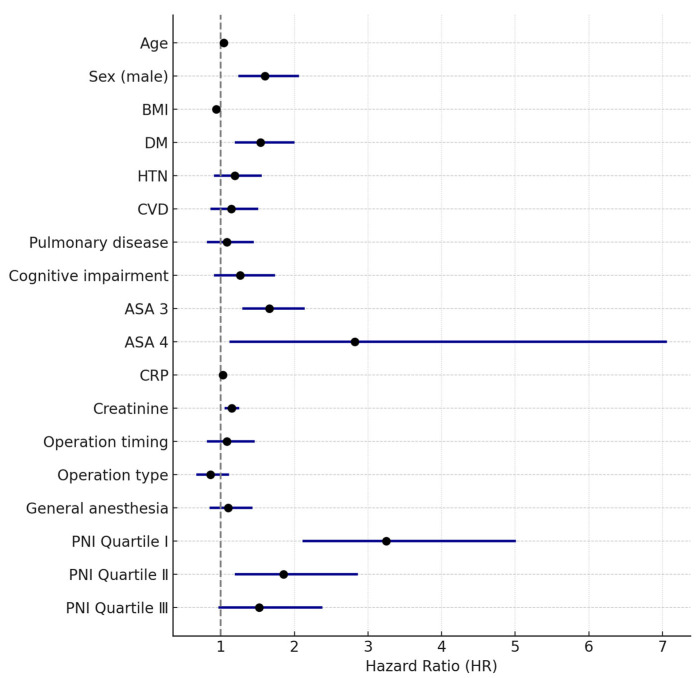
Forest plot of adjusted hazard ratios (HRs) for overall mortality based on multivariable Cox regression analysis.

For 1-year mortality, Cox regression showed significantly increased risk in Q1 and Q2 (Q1: HR 3.72, 95% CI 2.00–6.90, *p* < 0.001; Q2: HR 2.02, 95% CI 1.04–3.94, *p* = 0.038; Q4 as reference). Male sex (HR 1.62, 95% CI 1.10–2.39, *p* = 0.015), DM (HR 1.51, 95% CI 1.02–2.22, *p* = 0.039), ASA class 3 (HR 1.78, 95% CI 1.19–2.66, *p* = 0.005), and ASA class 4 (HR 3.60, 95% CI 1.10–11.80, *p* = 0.034) were significantly associated with higher 1-year mortality (Table 4 and Figure 3).

Additional secondary outcomes are summarized as follows: Lower PNI quartiles were associated with higher readmission rates (29.1% in Q1, 18.1% in Q2, 12.7% in Q3, and 10.8% in Q4) and longer postoperative hospital stays (13.76 days in Q1, 10.75 days in Q2, 10.34 days in Q3, and 8.81 days in Q4) (Table 2). In multivariable logistic regression analysis, patients in Q1 and Q2 were significantly associated with prolonged hospital stay (OR 1.97, 95% CI 1.44–2.71, *p* < 0.001) (Supplementary Table S1).

Although unadjusted rates of overall complications and delirium were higher in the lower PNI quartiles, these differences were not statistically significant after adjustment for covariates in multivariable analysis (overall complications: OR 1.20, 95% CI 0.86–1.67, *p* = 0.28; delirium: OR 1.20, 95% CI 0.87–1.67, *p* = 0.26; Supplementary Table S1). Similarly, rates of specific complications—including cardiovascular, pulmonary, neurologic events, and acute kidney injury—did not differ significantly across PNI quartiles (Table 2).

## 4. Discussion

In this retrospective analysis of 815 patients aged ≥65 years who underwent surgery for hip fracture, we observed a step-wise association between lower preoperative PNI and both 1-year and overall mortality. After adjustment of recognized geriatric and peri-operative risk factors including age, sex, BMI, ASA class, patients in the lowest PNI quartile had a three-fold higher hazard of death than those in the highest quartiles. This graded relationship extended to secondary outcomes, although less markedly: patients with lower PNI experienced longer hospitalization and more frequent readmissions, whereas rates of delirium and composite complications showed no significant differences between quartiles.

Most previous studies on the prognostic value of the prognostic nutritional index (PNI) have focused on elective surgical populations, particularly those with cancer or relatively preserved health [9,10,12,13]. In contrast, our study examined frail older adults with acute hip fracture, who are especially vulnerable to adverse postoperative outcomes and face substantial challenges in recovery, leading to significant socioeconomic burdens [1]. Although PNI has been associated with adverse postoperative outcomes in patients with hip fractures, studies specifically evaluating its correlation with overall mortality are limited [14,15]. Our findings suggest that PNI may have important prognostic value in acute orthopedic trauma, highlighting the need for further research to refine risk stratification and intervention strategies for this high-risk population.

Consequently, there is growing interest in identifying and managing high-risk subgroups within this population. Malnutrition is prevalent among patients with hip fracture and can adversely affect their functional status at discharge [16]. The PNI serves as an integrated marker of nutritional and immunological status. Hypoalbuminemia in these patients may reflect not only undernutrition but also systemic inflammation and the stress response, while lymphopenia may indicate immune senescence and diminished host defenses [17,18]. These physiological disturbances are further exacerbated by the prolonged catabolic state induced by trauma and surgical stress [19]. Given the high mortality following hip fracture surgery in older adults, identifying prognostic biomarkers or developing clinical scoring systems is imperative.

Prior studies using PNI for risk stratification have often suggested a cut-off value of approximately 45–50 [10,20]. However, applying a PNI cut-off of 45 in our cohort would classify approximately 80% of patients as high-risk. This aligns with prior research indicating a malnutrition prevalence of approximately 70% among patients with hip fractures [16]. Given the frailty of our cohort and potential influences such as diet and ethnicity on PNI, we stratified patients into quartiles rather than applying a fixed threshold. The observed stepwise increase in mortality and morbidity with decreasing PNI supports the utility of quartile-based stratification for refined risk assessment. Although frailty is a multifaceted construct encompassing physical function, cognition, and physiological reserve, the PNI may serve as a surrogate for its nutritional and immunological components—domains closely associated with postoperative outcomes.

Malnutrition is associated with osteoporosis, and a high prevalence of malnutrition has been observed in patients with pelvic fractures [21]. Functional status at discharge from pelvic fractures is a known predictor of mortality [22], and improvements in nutritional status during hospitalization have been linked to better recovery in functional capacity [23]. According to guidelines from the European Society for Clinical Nutrition and Metabolism, nutritional support and rehabilitation therapy are recommended for patients with pelvic fractures [24].

Thus, promoting physical and functional recovery through effective patient management is critical. Key strategies include postoperative rehabilitation, early nutritional support, and preoperative optimization [23,25,26,27,28,29]. However, it remains uncertain whether these strategies should be applied universally or targeted toward high-risk individuals. This question is especially relevant given the potential survival benefit of early surgery (within 48 h) for patients with hip fractures [30,31,32,33,34]. Avoiding unnecessary surgical delays is essential; however, when proper optimization measures are taken, surgery delayed beyond 48 h does not appear to increase mortality [35]. Thus, screening for high-risk patients and addressing modifiable factors preoperatively is vital [35]. In this context, PNI offers a simple, objective, and clinically useful assessment, as supported by our findings linking it to mortality risk.

This study has several limitations. Its retrospective design precludes the establishment of causal relationships and may be subject to missing data. To mitigate potential bias, we performed a multivariable regression analysis. Additionally, the single-center setting may limit the generalizability of the findings. As this study was conducted at a tertiary referral center with specialized perioperative protocols for geriatric hip fracture patients, there may be selection bias favoring individuals with better access to coordinated care. This could result in underestimation of mortality risk, particularly in lower PNI groups, compared with more heterogeneous care settings. Moreover, unmeasured variables such as cognitive impairment, pre-fracture mobility, and functional reserve were not available in our dataset and may act as residual confounders. The omission of these domains could bias the observed association between low PNI and mortality, potentially overestimating its prognostic strength. Finally, we did not directly compare the prognostic value of PNI with other established indices such as the Charlson Comorbidity Index or CONUT score, as these variables were either not associated with mortality in this setting or not routinely measured in all patients. Future studies should address their relative utility in hip fracture populations.

## 5. Conclusions

This retrospective study found that lower preoperative PNI was associated with higher mortality in older adults undergoing hip fracture surgery. Our findings suggest that PNI may serve as a valuable predictor of mortality in this high-risk population, although prospective validation is needed.

## Figures and Tables

**Table 1:** Patient characteristics and perioperative variables.

	Quartile I (n = 203)	Quartile II (n = 204)	Quartile III (n = 204)	Quartile IV (n = 204)	SMD
Demographic variables					
Age, years	80.86 ± 7.50	81.74 ± 7.38	80.42 ± 7.35	78.35 ± 7.33	0.240
Sex, male	65 (32.0)	64 (31.4)	49 (24.0)	42 (20.6)	0.158
BMI, kg/m^2^	21.12 ± 3.51	21.30 ± 3.57	22.23 ± 3.72	23.23 ± 3.57	0.338
DM	74 (36.5)	44 (21.6)	55 (27.0)	65 (31.9)	0.184
HTN	123 (60.6)	112 (54.9)	118 (57.8)	114 (55.9)	0.064
CVD	47 (23.2)	42 (20.6)	50 (24.5)	30 (14.7)	0.135
ASA					0.352
II	97 (47.8)	107 (52.5)	134 (65.7)	154 (75.5)	
III	103 (50.7)	96 (47.1)	69 (33.8)	48 (23.5)	
IV	3 (1.5)	1 (0.5)	1 (0.5)	2 (1.0)	
Operation-related variables					
Operation timing < 48 h	46 (22.7)	67 (32.8)	73 (35.8)	109 (53.4)	0.339
Operation type					
Arthroplasty	79 (38.9)	83 (40.7)	91 (44.6)	92 (45.1)	0.076
Osteosynthesis	124 (61.1)	121 (59.3)	113 (55.4)	112 (54.9)	
Anesthesia technique					0.107
General anesthesia	85 (41.9)	76 (37.3)	88 (43.1)	70 (34.3)	
Spinal anesthesia	118 (58.1)	128 (62.7)	116 (55.4)	134 (65.7)	
Laboratory variables					
PNI	31.61 ± 3.89	37.75 ± 0.98	41.08 ± 0.91	46.18 ± 3.18	3.151
WBC	8.71 ± 3.65	9.42 ± 3.52	10.06 ± 3.03	11.25 ± 4.12	0.374
Hemoglobin	9.87 ± 1.77	10.92 ± 1.65	11.64 ± 1.74	12.32 ± 1.47	0.810
CRP	4.24 ± 4.89	2.66 ± 3.76	1.43 ± 2.53	0.91 ± 1.85	0.532
Albumin; g/dL	2.74 ± 0.43	3.26 ± 0.23	3.52 ± 0.21	3.76 ± 0.28	1.797
Creatinine; mg/dL	1.31 ± 1.49	1.15 ± 1.34	1.00 ± 1.00	0.88 ± 0.71	0.205

Results are expressed as mean ± SD, n (%). SMD, standardized mean difference; BMI, body mass index; DM, diabetes mellitus; HTN, hypertension; CVD, cardiovascular disease; ASA, American Society of Anesthesiologists; WBC, white blood cell; CRP, C-reactive protein; PNI, prognostic nutritional index.

**Table 2:** Postoperative outcomes stratified by PNI quartiles.

	Quartile I (n = 203)	Quartile II (n = 204)	Quartile III (n = 204)	Quartile IV (n = 204)	SMD
Cardiovascular complication	10 (4.9)	4 (2.0)	9 (4.4)	7 (3.4)	0.091
Pulmonary complication	20 (9.9)	21 (10.3)	15 (7.4)	15 (7.4)	0.067
Neurologic complication	3 (1.5)	5 (2.5)	3 (1.5)	1 (0.5)	0.084
Acute kidney injury	27 (13.3)	15 (7.4)	18 (8.8)	11 (5.4)	0.147
Delirium	53 (26.1)	62 (30.4)	55 (27.0)	26 (12.7)	0.222
Overall complication	93 (43.3)	87 (44.2)	74 (37.2)	49 (24.1)	
Readmission	59 (29.1)	37 (18.1)	26 (12.7)	22 (10.8)	0.260
Hospital stay, days	13.76 ± 21.85	10.75 ± 10.52	10.34 ± 13.04	8.81 ± 6.90	0.178
1-year mortality	54 (26.6)	29 (14.2)	14 (6.9)	13 (6.4)	0.325
Overall mortality	115 (56.7)	74 (36.3)	55 (27.0)	31 (15.2)	0.499

Results are expressed as mean ± SD, n (%). PNI, prognostic nutritional index; SMD, standardized mean difference.

**Table 3:** Cox regression analyses of risk factors associated with overall mortality.

		Univariate			Multivariate	
	HR	95% CI	*p*	HR	95% CI	*p*
Age	1.04	1.02–1.06	<0.001	1.04	1.02–1.06	<0.001
Sex (male)	1.53	1.15–2.04	0.004	1.60	1.24–2.06	<0.001
BMI	0.94	0.91–0.97	<0.001	0.94	0.91–0.97	<0.001
DM	1.48	1.12–01.96	0.005	1.54	1.19–2.00	<0.001
HTN	1.19	0.91–1.56	0.215			
CVD	1.14	0.86–1.51	0.376			
Pulmonary disease	1.08	0.81–1.45	0.597			
Cognitive impairment	1.26	0.91–1.74	0.165			
ASA						
1 or 2	1.00	Reference		1.00	Reference	
3	1.69	1.29–2.21	<0.001	1.66	1.29–2.14	<0.001
4	2.73	1.07–6.99	0.036	2.82	1.12–7.06	0.027
CRP	1.02	1.99–1.05	0.203	1.03	1.00–1.06	0.035
Creatinine	1.15	1.06–1.25	0.001	1.15	1.05–1.25	0.001
Operation timing	1.08	0.81–1.46	0.592			
Operation type	0.86	0.67–1.11	0.247			
General anesthesia	1.10	0.85–1.43	0.451			
PNI Quartile I	2.71	1.58–4.64	<0.001	3.25	2.11–5.01	<0.001
PNI Quartile II	1.57	0.93–2.64	0.091	1.85	1.19–2.86	0.006
PNI Quartile III	1.39	0.87–2.23	0.170	1.52	0.97–2.38	0.065
PNI Quartile IV	1.00	Reference		1.00	Reference	

HR, hazard ratio; CI, confidence interval; BMI, body mass index; DM, diabetes mellitus; HTN, hypertension; CVD, cardiovascular disease; ASA, American Society of Anesthesiologists; CRP, C-reactive protein; PNI, prognostic nutritional index.

**Table 4:** Cox regression analyses of risk factors associated with 1-year survival.

		Univariate			Multivariate	
	HR	95% CI	*p*	HR	95% CI	*p*
Age	1.02	0.99–1.04	0.270			
Sex (male)	1.40	0.89–2.19	0.143	1.62	1.10–2.39	0.015
BMI	0.95	0.90–1.01	0.109			
DM	1.58	1.02–2.45	0.038	1.51	1.02–2.22	0.039
HTN	0.91	0.59–1.41	0.684			
CVD	1.00	0.63–1.59	0.988			
Pulmonary disease	0.99	0.63–1.56	0.960			
Cognitive impairment	1.13	0.67–1.93	0.645			
ASA						
1 or 2	1.00	Reference		1.00	Reference	
3	1.70	1.10–2.63	0.017	1.78	1.19–2.66	0.005
4	3.38	0.98–77.71	0.054	3.60	1.10–11.80	0.034
CRP	1.00	0.96–1.05	0.834			
Creatinine	1.14	1.01–1.29	0.032			
Operation timing	1.56	0.95–2.56	0.080			
Operation type	0.94	0.63–1.39	0.754			
General anesthesia	1.39	0.91–2.11	0.130			
PNI Quartile I	2.02	0.88–4.63	0.097	3.72	2.00–6.90	<0.001
PNI Quartile II	1.24	0.55–2.80	0.606	2.02	1.04–3.94	0.038
PNI Quartile III	0.78	0.35–1.74	0.544	1.02	0.48–2.17	0.962
PNI Quartile IV	1.00	Reference		1.00	Reference	

HR, hazard ratio; CI, confidence interval; BMI, body mass index; DM, diabetes mellitus; HTN, hypertension; CVD, cardiovascular disease; ASA, American Society of Anesthesiologists; CRP, C-reactive protein; PNI, prognostic nutritional index.

## Data Availability

The datasets used and/or analyzed during the current study are available from the corresponding author on reasonable request.

## Supplementary Materials

The following supporting information can be downloaded at: https://www.mdpi.com/article/10.3390/medicina61081376/s1.

## Abbreviations

The following abbreviations are used in this manuscript:

PNI	Prognostic Nutritional Index
BMI	Body Mass Index
ASA	American Society of Anesthesiologists
Hb	Hemoglobin
CRP	C-reactive Protein
CI	Confidence Interval
HR	Hazard Ratio
OR	Odds Ratio
DM	Diabetes Mellitus
