# Supplementary material for: Association of Prognostic Nutritional Index and Mortality in Older Adults Undergoing Hip Fracture Surgery: A Retrospective Observational Study at a Single Large Center
Source: Medicina (Kaunas). 2025 Jul 30;61(8):1376. doi: 10.3390/medicina61081376 (PMC12388270; doi:10.3390/medicina61081376)
Supplement: Supplementary file 1 [file medicina-61-01376-s001.zip › medicina-3745772-supplementary.pdf]

Supplementary Table S1. Logistic regression analysis of surgical outcomes stratified by preoperative PNI

	Univariate			Multivariate		
	**OR**	**95% CI**	***P***	**OR**	**95% CI**	***P***
**Hospital stay, days**						
PNI Quartiles Ⅲ, Ⅳ	1.00					
PNI Quartiles Ⅰ, Ⅱ	2.06	1.51–2.82	< 0.001	1.97	1.44–2.71	< 0.001
**Overall complication**						
PNI Quartiles Ⅲ, Ⅳ	1.00					
PNI Quartiles Ⅰ, Ⅱ	1.76	1.32–2.35	< 0.001			
**Delirium**						
PNI Quartiles Ⅲ, Ⅳ	1.00					
PNI Quartiles Ⅰ, Ⅱ	1.69	1.22–2.34	0.002			

OR, odds ratio; CI, confidence interval; PNI, prognostic nutritional index
